# Mitochondrial genome of the fluke pond snail, *Austropeplea* cf.* brazieri* (Gastropoda: Lymnaeidae)

**DOI:** 10.1186/s13071-024-06358-7

**Published:** 2024-07-02

**Authors:** Tanapan Sukee, Anson V. Koehler, Bonnie L. Webster, Charles G. Gauci, Conor E. Fogarty, Winston F. Ponder, Robin B. Gasser, Neil D. Young

**Affiliations:** 1 Melbourne Veterinary School, Faculty of Science, The University of Melbourne, Victoria, Australia; 2 Natural History Museum, London, UK; 3 Australian Museum, Sydney, NSW Australia

**Keywords:** *Austropeplea *, Australia, *Fasciola hepatica*, Snail, Intermediate host, Mitochondrial genome

## Abstract

**Background:**

Lymnaeid snails of the genus *Austropeplea* are an important vector of the liver fluke (*Fasciola hepatica*), contributing to livestock production losses in Australia and New Zealand. However, the species status within *Austropeplea* is ambiguous due to heavy reliance on morphological analysis and a relative lack of genetic data. This study aimed to characterise the mitochondrial genome of *A.* cf. *brazieri*, an intermediate host of liver fluke in eastern Victoria.

**Methods:**

The mitochondrial genome was assembled and annotated from a combination of second- and third-generation sequencing data. For comparative purposes, we performed phylogenetic analyses of the concatenated nucleotide sequences of the mitochondrial protein-coding genes, cytochrome *c* oxidase subunit 1 and 16S genes.

**Results:**

The assembled mt genome was 13,757 base pairs and comprised 37 genes, including 13 protein-coding genes, 22 transfer RNA genes and 2 ribosomal RNA genes. The mt genome length, gene order and nucleotide compositions were similar to related species of lymnaeids. Phylogenetic analyses of the mt nucleotide sequences placed *A.* cf. *brazieri* within the same clade as *Orientogalba ollula* with strong statistical supports. Phylogenies of the *cox*1 and 16S mt sequences were constructed due to the wide availability of these sequences representing the lymnaeid taxa. As expected in both these phylogenies, *A.* cf. *brazieri* clustered with other *Austropeplea* sequences, but the nodal supports were low.

**Conclusions:**

The representative mt genome of *A.* cf. *brazieri* should provide a useful resource for future molecular, epidemiology and parasitological studies of this socio-economically important lymnaeid species.

**Graphical abstract:**

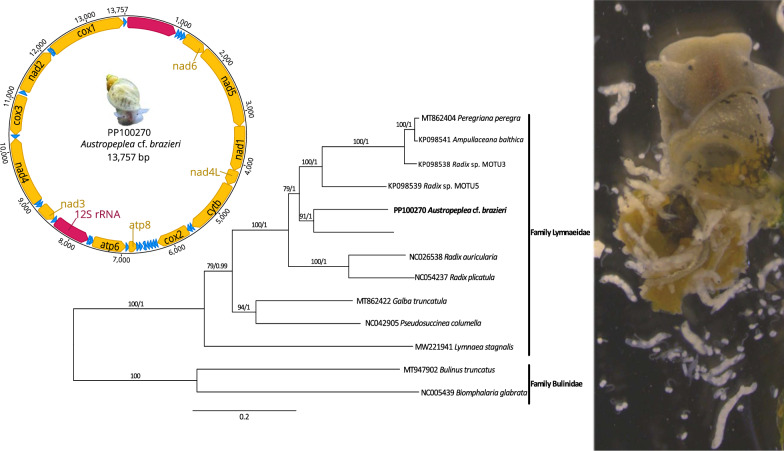

**Supplementary Information:**

The online version contains supplementary material available at 10.1186/s13071-024-06358-7.

## Background

Freshwater gastropods of the family Lymnaeidae, known commonly as pond snails, are a diverse group with a worldwide distribution. Many lymnaeid taxa are vectors of socio-economically significant parasitic trematodes (flukes) [1]. In Australia, where livestock production is impacted by fascioliasis, a disease caused by the liver fluke *Fasciola hepatica* [2–4], lymnaeid snails of the genus *Austropeplea* are considered the most important native intermediate hosts of this parasitic trematode [5, 6].

*Austropeplea* is a semi-amphibious lymnaeid, occurring in freshwater habitats, such as ponds, streams and wetlands in south-eastern Australia and in New Zealand [7]. Currently, this genus is proposed to comprise at least four species and two subgenera [7], although the number of species still requires verification. In the past, 23 Australian and New Zealand lymnaeid species–group names were synonymised as *Austropeplea tomentosa* on the basis that their morphological variation related to phenotypic plasticity, induced by environmental factors [8]. Recently, however, it was proposed that at least three species of *Austropeplea* are endemic to south-eastern Australia (*A. brazieri* and *A. subaquatilis)*, including Tasmania (*A. huonensis*) [7] and that *A. tomentosa* is exclusive to New Zealand on the basis of combined morphological and molecular investigations [9]. However, current molecular systematic studies of the Lymnaeidae conducted to date have utilised DNA sequence data only for a very small number of genetic markers in nuclear DNA (particularly the internal transcribed spacers of rDNA) and in mitochondrial (mt) DNA (*cox*1 and 16S genes) [10–14]. Thus, conclusions regarding species status and phylogenetic position within the family Lymnaeidae are likely restricting. Nonetheless, these studies have underpinned the next step, which is to use genomic data sets to ‘barcode’ species or taxa (the majority of which are presently defined using morphological data) for robust analyses of relationships among them.

In the present study, we characterised the first complete mitochondrial genome of a key representative of the Lymnaeidae. The focus here is on a taxon we refer to as *Austropeplea* cf. *brazieri*, which is a fluke pond snail that is distributed in south-eastern Australia [7] and inferred to be the predominant intermediate host of liver fluke affecting livestock production in this region [5]. The designation of this taxon indicates its indeterminate species status. The mt genome provided in this study will serve as a reference mt genome for future taxonomic, phylogenetic and ecological work on key snail vectors of parasitic trematodes.

## Methods

### Sample collection

Specimens of *A.* cf. *brazieri* were collected from a roadside irrigation channel in Werribee South, Victoria, Australia (latitude −37.944706, longitude 144.698857) and maintained in aquaria within a designated laboratory in the Department of Veterinary Biosciences, The University of Melbourne, Victoria, Australia. Individual snails were de-shelled, thoroughly washed in phosphate-buffered saline (PBS, pH 7.0), snap frozen in liquid nitrogen and stored at − 80 °C prior to DNA isolation.

### DNA isolation, library construction and sequencing

The Nanobind Tissue Kit (PacBio, Menlo Park, CA, USA) was used to isolate high molecular weight genomic DNA from a single adult *A.* cf. *brazieri*. The quality of isolated DNA was evaluated using an Agilent 4200 TapeStation system (Thermo Fisher Scientific Waltham, MA, USA) and Genomic DNA ScreenTape (Thermo Fisher Scientific). The Ligation Sequencing Kit (SQK-LSK109; Oxford Nanopore Technologies) was used to construct a genomic DNA library following the manufacturer’s protocol. The library was then sequenced using a MinION sequencer (Oxford Nanopore Technologies). The flow cell used to sequence the library was washed using the Flow Cell Wash Kit (EXP-WSH003; Oxford Nanopore Technologies) and re-used to re-sequence the same DNA library. Base calling from raw FAST5 reads was done using the program Guppy v.5 (Oxford Nanopore Technologies) and saved in the FASTQ format [15].

### Assembly and isolation of the mitochondrial genome

De novo assembly of the long-read sequences was performed using FLYE v.2.6 [16] with the -nano-raw option, and errors were corrected using medaka_consensus in the Medaka package v.0.10.0 (https://github.com/nanoporetech/medaka). The long-read sequence data were then mapped back to the assembled mitochondrial (mt) genome using Minimap2 v.2.0 [17], and mosdepth [18] was used to estimate genome coverage.

**Fig. 1:**
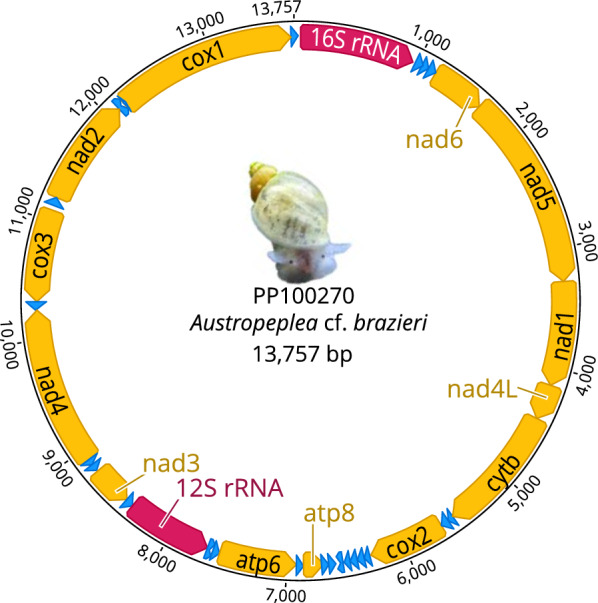
Reference mitochondrial genome of *Austropeplea* cf. *brazieri* (GenBank accession no. PP100270). The direction of gene transcription is shown with an arrow. Long (16S) and short (12S) ribosomal RNA subunits are shown in red and protein-encoding genes are shown in yellow

Initial annotation of tRNA, rRNA and protein-encoding gene regions was performed on the MITOS webserver [19] using the invertebrates mt genetic code (https://www.ncbi.nlm.nih.gov/Taxonomy/Utils/wprintgc.cgi; translation_table 5). Protein-coding genes were further curated in the program Geneious v.11.1.5 [20] using open reading frames (ORF) and published lymnaeid mt genomes as a guide (Table 1). The complete mt genome sequence was deposited in the GenBank database under accession no. PP100270 (Fig. 1). Raw sequence data are available from Sequence Read Archive (SRA) under accession no. SAMN39324652 with NCBI BioProject accession no. PRJNA1088272.

**Table 1:** Mitochondrial genome sequences of snail species or strains used in the present study, with GenBank accession numbers and references listed

GenBank accession number	Species − ‘strain’	Length (bp)	G + C content (%)	References
PP100270	*Austropeplea* cf. *brazieri*	13,757	26.71	Present study
KP098538	*Radix* sp. “MOTU3”	13,963	28.73	[21]
KP098539	*Radix sp.—*‘MOTU5’	13,832	25.98	[21]
KP098541	*Radix balthica*	13,983	28.68	[21]
MT862404	*Peregriana peregra*	14,023	28.44	Direct submission
MT862422	*Galba truncatula*	13,855	26.06	Direct submission
MT947902	*Bulinus truncatus*	13,767	24.29	Direct submission
MW221941	*Lymnaea stagnalis*	13,834	28.13	Direct submission
NC005439	*Biomphalaria glabrata*	13,670	25.37	[22]
NC018536	*Orientogalba ollula*	13,768	27.32	[23]
NC026538	*Radix auricularia*	13,745	29.31	[21]
NC042905	*Pseudosuccinea columella*	13,757	26.66	Direct submission
NC054237^a^	*Radix plicatula*	13,751	29.69	[24]

^a^The name of the organism listed under this GenBank accession number is *Ampullaceana lagotis*, but its referenced publication [24] clearly states that this mitochondrial sequence belongs to *Radix plicatula*; therefore, we have listed it in this study as such

### Whole mt genome and single loci comparative analyses

The complete mt genome of *A*. cf. *brazieri* was compared with the available reference mt genomes of other lymnaeids from the NCBI database (Table 1). We used the key words ‘Lymnaeidae + mitochondrial + genome’ and ‘Lymnaeidae + mitochondrion + genome’ in the ‘Nucleotide’ database (20 June 2023). The *Bulinus truncatus* and *Biomphalaria glabrata* (family Planorbidae) were used as outgroups. The comparison was performed with progressiveMauve v.2.4.0 [25] using the settings -hmm-identity = 0.95 and -island-gap-size = 10. Mitochondrial protein-coding genes were subsequently extracted and aligned as separate nucleotide coding sequences or inferred amino acid sequences using MUSCLE v.3.7 [26] alignment tool. The optimal nucleotide substitution model for each aligned sequence was determined using ModelTest-NG v.0.1.6 [27]. The aligned sequences were then subjected to phylogenetic analysis using Bayesian inference (BI) or maximum likelihood (ML) methods employing Monte Carlo Markov chain analysis in the program MrBayes v.3.2.2 [28] and IQ-tree v.2.2.2.7 [29], respectively. For the BI analysis, posterior probabilities (PP) were calculated using the optimal nucleotide substitution model (*cox*1 and 16S rRNA: GTR + I + G), generating 2,000,000 trees and sampling every 200th tree until potential scale reduction factors for each parameter approached 1. The initial 25% of trees were discarded as burn-in, and the others were used to construct a majority rule tree. Maximum likelihood trees and bootstrap (BS) supports were inferred using the optimal nucleotide substitution models and using the option ‘-B 10000 -bnni -minsup 0.5 -bi 1000’. The initial 10% of the 10,000 trees were discarded as burn-in, and the others were used to construct a majority rule tree. Phylogenetic trees were rendered and annotated using ggtree v.1.10.5 [30] in R v.4.3.1 (http://www.R-project.org/).

Due to the wide availability of mt *cox*1 and 16S rRNA sequence data for the family Lymnaeidae, we performed phylogenetic analyses of *A.* cf. *brazieri* and other lymnaeids based on these individual genes. Mitochondrial *cox*1 and 16S rRNA sequence data were downloaded from NCBI nucleotide sequence database (7 June 2023; Supplementary Table S1), with *B. truncatus* (gene ID 70630849) and *Bi. glabrata* (gene ID 2746309) (family Planorbidae) as outgroups. For the *cox*1 dataset, the key words ‘Lymnaeidae *cox*1’, ‘Lymnaeidae coi’, ‘Lymnaeidae cytochrome c oxidase subunit I’ and ‘Lymnaeidae cytochrome c oxidase subunit 1’ were used (Supplementary Table S1). For the 16S rRNA dataset, the key word ‘Lymnaeidae 16S’ was used. For each dataset, identical nucleotide sequences were removed using CD-HIT-EST v.4.6 [31]. Sequences incorrectly placed in the Lymnaeidae family or containing too few nucleotide sequences were also removed. The remaining sequences were aligned using MUSCLE. The optimal nucleotide substitution model for aligned sequences was then assessed using the program ModelTest-NG v.0.1.6 [27]. The aligned sequences were concatenated and then subjected to phylogenetic analysis using BI or ML methods as described above.

**Fig. 2:**
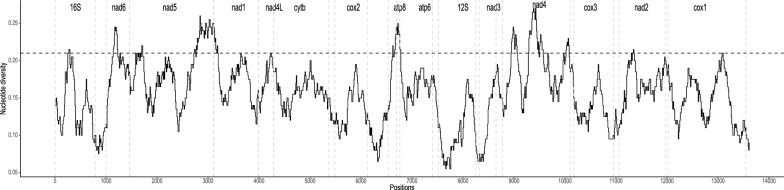
Sliding window analyses of the tRNA and concatenated protein-coding nucleotide sequences of *Austropeplea* cf. *brazieri* and *Orientogalba ollula* (*Galba pervia*, GenBank accession no. NC018536) mitochondrial genomes. Gene boundaries are indicated by vertical dotted lines. The horizontal dotted line indicates the average nucleotide diversity across both mitochondrial genomes

Comparison of nucleotide diversity patterns between the aligned mt protein-coding regions of *A*. cf. *brazieri* and protein-coding regions of the reference mt genome of *Orientogalba ollula* (= *Galba pervia*, GenBank accession no. NC018536) was performed using a sliding window analysis (Fig. 2). A sliding window analysis of nucleotide diversity (steps of 10 bp over 200-bp windows) was performed for each pairwise-alignment of concatenated genes using the PopGenome package [32] in R. For each comparison, nucleotide diversity values were plotted using the R package ggplot2 [33].

## Results and discussion

The average depth of coverage of long-reads mapped to the mt genome of *A.* cf. *brazieri* was 463.09 (standard deviation = 30.56). For the short reads, the average depth of coverage was 958.85 (standard deviation 95.52). The mt genome of *A*. cf. *brazieri* (GenBank accession number PP100270) is circular and spans 13,757 base pairs (bp) in length (equivalent to 13.8 kb) which falls within range (13.6–14.1 kb) of heterobranch gastropod mt genomes sequenced so far [34]. We identified 37 genes which included 13 protein-coding genes, 22 transfer RNA genes and 2 ribosomal RNA genes (Fig. 1; Table 2). In most instances, start and stop codons were consistent with those of the mt genomes of most molluscs [34] and other invertebrates characterised to date [35].

**Table 2:** Mitochondrial genes of *Austropeplea* cf. *brazieri* and their locations, GC contents, lengths, start/stop codons and direction of the protein-coding gene transcription

Gene designations	Location (start/end)	Length (bp)	Start/stop codons	Transcription direction
*rrnL*	1/986	962	NA	Forward
tRNA-L1(tag)	988/1051	64	NA	Forward
tRNA-P(tgg)	1045/1107	63	NA	Forward
tRNA-A(tgc)	1107/1170	64	NA	Forward
*nad*6	1171/1629	459	ATA/TAA	Forward
*nad*5	1631/3277	1647	ATA/TAG	Forward
*nad*1	3279/4154	876	ATT/TAA	Forward
*nad*4L	4155/4452	298	TTG/TAA	Forward
*cytb*	4453/5535	1083	ATT/TAA	Forward
tRNA-D(gtc)	5536/5587	53	NA	Forward
tRNA-F(gaa)	5588/5650	62	NA	Forward
*cox*2	5651/6294	643	TTG/TAA	Forward
tRNA-Y(gta)	6296/6345	50	NA	Forward
tRNA-W(tca)	6346/6405	60	NA	Forward
tRNA-C(gca)	6410/6468	59	NA	Forward
tRNA-G(tcc)	6470/6522	53	NA	Forward
tRNA-H(gtg)	6525/6582	58	NA	Forward
tRNA-Q(ttg)	6649/6591	59	NA	Reverse
tRNA-L2(taa)	6701/6650	53	NA	Reverse
*atp*8	6853/6702	165	ATC/TAA	Reverse
tRNA-N(gtt)	6917/6854	64	NA	Reverse
*ATP*6	7557/6917	641	TTG/TAA	Reverse
tRNA-R(tcg)	7620/7558	63	NA	Reverse
tRNA-E(gaa)	7672/7621	52	NA	Reverse
*rrnS*	8388/7673	717	NA	Reverse
tRNA-M(cat)	8457/8389	69	NA	Reverse
*nad*3	8797/8460	340	ATA/TAA	Reverse
tRNA-S2(tga)	8866/8804	63	NA	Reverse
tRNA-S1(gct)	8863/8917	55	NA	Forward
*nad*4	8918/10,243	1326	ATT/TAG	Forward
tRNA-T(tgt)	10,311/10,244	68	NA	Reverse
*cox*3	11,092/10,313	780	ATG/TAA	Reverse
tRNA-I(gat)	11,133/11,197	65	NA	Forward
*nad*2	11,198/12,121	907	ATT/TAG	Forward
tRNA-K(ttt)	12,102/12,180	79	NA	Forward
*cox*1	12,192/13,685	1493	ATT/TAA	Forward
tRNA-V(tac)	13,684/13,744	60	NA	Forward

The nucleotide composition within *A*. cf. *brazieri* mt genome was A + T biased (*A* = 36.9%, *C* = 11.9%, *G* = 12.8% and *T* = 38.5%). This A + T nucleotide composition bias has also been observed in other lymnaeid species sequenced to date, including *Pseudosuccinea columella* (73.3%) *Orientogalba ollula* (as *Galba pervia*) (72.69%), *Radix auricularia* (70.7%) and *Radix plicutula* (70.3%). The tRNAs (Table 2) were inferred to have a canonical structure. Two copies of a serine and a leucine tRNA were encoded, and all tRNAs were predicted to have DHU and TψC arms, except for tRNA-G(tcc) (without a TψC arm), tRNA-S1(gct) (without a DHU arm) and tRNA-S2(tga) (without a DHU arm).

The arrangement of genes within the mt genome of *A*. cf. *brazieri* are identical to those of *O. ollula* [23]. Future studies sequencing the complete mt genomes of additional taxa related to *Austropeplea* could potentially reveal alternative gene arrangements. The Heterobranchia, a group which *Austropeplea* belongs to, has been found to display the most variable gene arrangement among the Gastropoda [36].

**Fig. 3:**
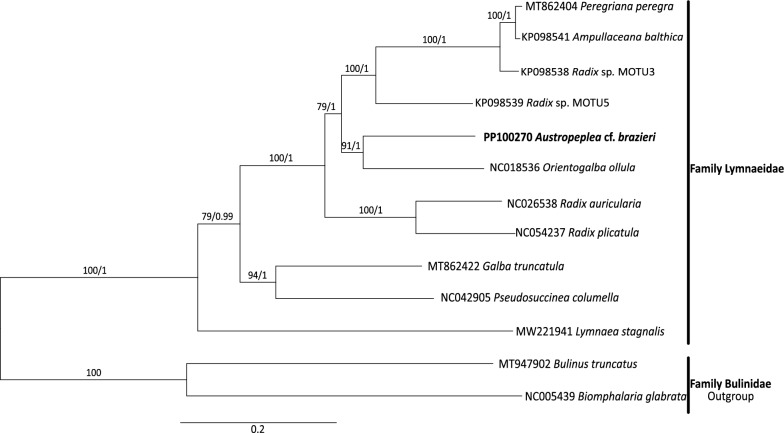
Phylogenetic relationship of *Austropeplea* cf*. brazieri* with other representative lymnaeid snails. *Biomphalaria glabrata* and *Bulinus truncatus* (family Planorbidae) are the outgroups (Table 1). A phylogeny was inferred from concatenated nucleotide sequences derived from 12 mitochondrial protein-encoding genes using Bayesian inference (BI) and maximum likelihood (ML) analyses. Bootstrap (BS) support for the ML and posterior probability (PP) of the BI analyses are indicated at each node of the tree. The scale bar indicates phylogenetic distance in substitutions per site. The updated nomenclature is based on Supplementary Table S2

Phylogenetic analyses (ML and BI) of the full-length mtDNA resulted in a phylogenetic tree with robust statistical supports (Fig. 3). There were good supports (BS/PP = 91/1) for the close relationship between *A.* cf. *brazieri* and *O. ollula* (as *Galba pervia*), originally described from eastern China [37]. This relationship, in addition to the clades comprising *Radix* and *Ampullaceana*, is consistent with the phylogeny based on the combined mt and nuclear sequence dataset [38]. However, with only 11 full-length lymnaeid mt genomes sequenced to date (including this study) out of around 175 described species, the inadequate representation of taxa within the current phylogenetic tree prevents any further interpretation.

**Fig. 4:**
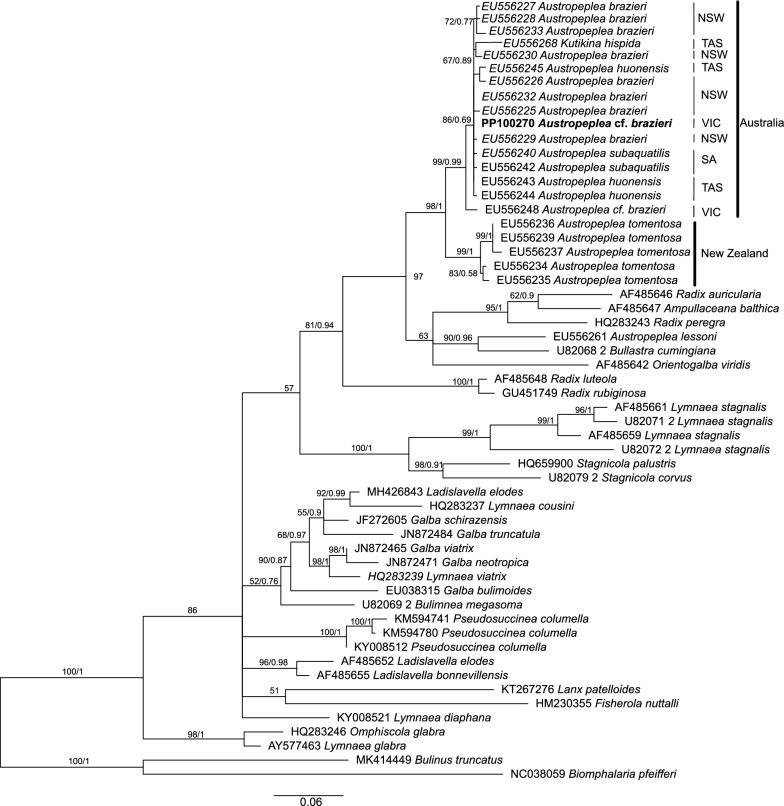
Phylogenetic relationship of *Austropeplea* cf. *brazieri* with other representative lymnaeid snails inferred on the basis of an analysis of the aligned partial mitochondrial 16S rRNA gene sequences by Bayesian inference (BI) and maximum likelihood (ML) using *Biomphalaria pfeifferi* and *Bulinus truncatus* (family Planorbidae) outgroups. Bootstrap (BS) support for the ML and posterior probability (PP) of the BI analyses are indicated at each node of the tree. The partial mitochondrial 16S rRNA gene sequence of *A.* cf. *brazieri* sequenced here is denoted in bold type. The updated nomenclature is based on Supplementary Table S2. The scale bar indicates phylogenetic distance in substitutions per site. *NSW* New South Wales, *TAS *Tasmania, *Vic* Victoria

**Fig. 5:**
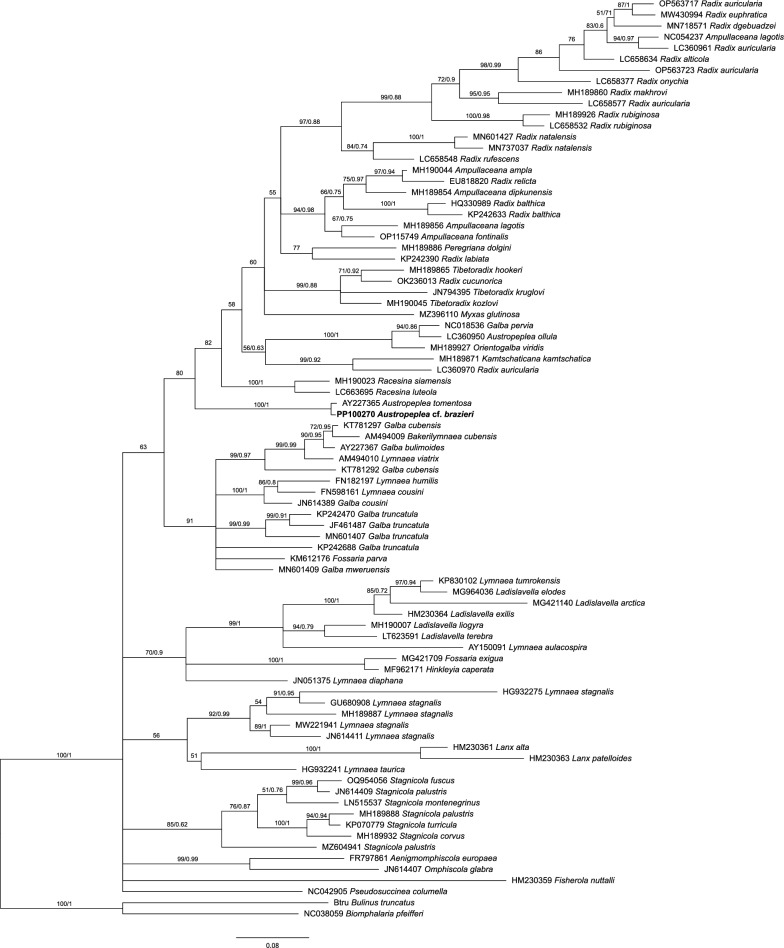
Phylogenetic relationship of *Austropeplea* cf. *brazieri* with other representative lymnaeid snails inferred on the basis of an analysis of the aligned partial *cox*1 mitochondrial gene sequences by Bayesian inference (BI) and maximum likelihood (ML) using *Biomphalaria pfeifferi* and *Bulinus truncatus* (family Planorbidae) as outgroups. Bootstrap (BS) support for the ML and posterior probability (PP) of the BI analyses are indicated at each node of the tree. The partial *cox*1 gene sequence of *A.* cf. *brazieri* sequenced here is denoted in bold type. The scale bar indicates phylogenetic distance (in substitutions per site)

We also assessed the phylogenetic relationship between *A.* cf. *brazieri* with other lymnaeids using the mitochondrial *cox*1 and 16S genes due to the wide availability of these sequences within the Lymnaeidae (Figs. 4, 5, respectively). The phylogenetic tree comprising 83 *cox1* sequences placed *A.* cf. *brazieri* in the same clade as *A. tomentosa* (GenBank AY227365) with strong support (BS/PP = 100/1). Since *A. tomentosa* is the only other species of *Austropeplea* with the *cox*1 gene sequenced, future studies containing additional *cox*1 sequences of this genus could determine the utility of this marker for resolving phylogenetic relationships within *Austropeplea*, although, at the genus level, the position of *Austropeplea* with other lymnaeid genera was ambiguous based on the *cox*1 gene. Similarly, the mt 16S phylogeny comprised 19 sequences (Fig. 5) and *A.* cf. *brazieri* fell within the Australian *A. tomentosa* group with low statistical support from both the ML and BI analyses. The lack of resolution within the current 16S phylogeny and the incongruences between this marker and other genetic regions [9] suggest that this topology may not accurately reflect the phylogenetic relationship of *Austropeplea*.

## Conclusions

Future studies to characterise the mt genomic sequences of the lymnaeids endemic to Australasia should allow some of the taxonomic ambiguities within this group to be addressed. Furthermore, such genomic datasets would provide greater insight into the phylogeny among the Australian lymnaeids and their relationships with other pond snails occurring worldwide. In conclusion, this study presented the first complete mt genome of *A.* cf. *brazieri*, which serves as a valuable resource for future molecular, epidemiological and ecological studies of this and related socio-economically important lymnaeid species.

### Supplementary Information

Additional file 1: Table S1. Partial *cox*1 gene and 16S trees sequences used in the present study, with GenBank accession numbers, description of taxon and references listed; Supplementary Table S2. The updated nomenclature used in the phylogenetic tree of the current study and the previous species names corresponding to each of the GenBank accession numbers.

## Data Availability

All relevant data are included in the article.
