# Supplementary material for: Mitochondrial genome of the fluke pond snail, Austropeplea cf. brazieri (Gastropoda: Lymnaeidae)
Source: Parasit Vectors. 2024 Jul 2;17:283. doi: 10.1186/s13071-024-06358-7 (PMC11218368; doi:10.1186/s13071-024-06358-7)
Supplement: Supplementary file 1 — Additional file 1: Table S1. Partial cox1 gene and 16S trees sequences used in the present study, with GenBank accession numbers, description of taxon and references listed; Supplementary Table S2. The updated nomenclature used in the phylogenetic tree of the current study and the previous species names corresponding to each of the GenBank accession numbers. [file 13071_2024_6358_MOESM1_ESM.docx]

**Supplementary Table S1.** Partial *cox*1 gene and 16S trees sequences used in the present study, with GenBank accession numbers, description of taxon and references listed.

| Accession no. | Description of taxon |
| --- | --- |
| AY227365 | *Austropeplea tomentosa* |
| MG421709 | *Fossaria exigua* voucher PPM032 |
| MF962171 | *Hinkleyia caperata* isolate NH1 |
| AY150091 | *Lymnaea aulacospira* isolate LA |
| MH190007 | *Ladislavella liogyra* voucher Mlym495/6 |
| LT623591 | *Ladislavella terebra* specimen voucher SNSD:Moll:52850 |
| MG421140 | *Ladislavella arctica* voucher 10PROBE-105440 |
| HM230364 | *Ladislavella exilis* isolate StumpLake |
| KP830102 | *Lymnaea tumrokensis* voucher Mlym-3 |
| MG964036 | *Ladislavella elodes* isolate SPRLS1 |
| MT947902 | *Bulinus truncatus* |
| NC038059 | *Biomphalaria pfeifferi* |
| HM230361 | *Lanx alta* isolate Amacher |
| HM230363 | *Lanx patelloides* isolate McCloud |
| FR797861 | *Aenigmomphiscola europaea* voucher MTD Moll S1151 |
| JN614407 | *Omphiscola glabra* isolate OG21 |
| HM230359 | *Fisherola nuttalli* isolate Bancroft |
| HG932275 | *Lymnaea stagnalis* voucher SNSD Tissue WL 8881 |
| MZ604941 | *Stagnicola palustris* haplotype St.p |
| LN515537 | *Stagnicola montenegrinus* mitochondrial partial *cox*1 |
| OQ954056 | *Stagnicola fuscus* x *Stagnicola palustris* isolate LYM-*cox*1 |
| MH189932 | *Stagnicola corvus* voucher Mlym123 |
| MH189888 | *Stagnicola palustris* voucher Mlym52 |
| KP070779 | *Stagnicola turricula* isolate Str12 |
| MH189860 | *Radix makhrovi* voucher Mlym35 |
| LC658577 | *Radix auricularia* L1503 |
| MH189926 | *Radix rubiginosa* voucher Mlym108 |
| LC658532 | *Radix rubiginosa* L1280 |
| LC658377 | *Radix onychia* L111 |
| OP563723 | *Radix auricularia* isolate D3 |
| NC.054237 | *Ampullaceana lagotis* voucher ncuwxp-nc-201901 |
| LC360961 | *Radix auricularia* haplotype: Ra03 |
| LC658634 | *Radix alticola* L1412 |
| MN718571 | *Radix dgebuadzei* voucher Mlym686/2 |
| OP563717 | *Radix auricularia* isolate B3 |
| MW430994 | *Radix euphratica* isolate Iran snail 9 |
| MH189871 | *Kamtschaticana kamtschatica* voucher Mlym44/4 |
| LC360970 | *Radix Auricularia* haplotype: Ra12 |
| HQ330989 | *Radix balthica* clone CHDO1-CHC01-18SSNO1-SKR01-FVI03 |
| KP242633 | *Radix balthica* isolate JRC1 |
| MH189854 | *Ampullaceana dipkunensis* voucher Mlym12 |
| MH190044 | *Ampullaceana ampla* voucher Mlym651 |
| EU818820 | *Radix relicta* isolate 5416 |
| MH189856 | *Ampullaceana lagotis* voucher Mlym13 |
| OP115749 | *Ampullaceana fontinalis* voucher PS3_Donetsk |
| MH189927 | *Orientogalba viridis* voucher Mlym109 |
| NC_018536 | *Galba pervia* |
| LC360950 | *Austropeplea ollula* haplotype: Ao01 |
| LC658548 | *Radix rufescens* L1411 |
| MN601427 | *Radix natalensis* voucher UGSB 23483 |
| MN737037 | *Radix natalensis* voucher Mlym743 |
| PP100270 | *Austropeplea brazieri* |
| JN794395 | *Tibetoradix kruglovi* isolate 10065 |
| MH189865 | *Tibetoradix hookeri* voucher Mlym38 |
| MH190045 | *Tibetoradix kozlovi* voucher Mlym685/1 |
| OK236013 | *Radix cucunorica* isolate RCQH0002 |
| MH190023 | *Racesina siamensis* voucher Mlym601 |
| LC663695 | *Racesina luteola* L1422 |
| MZ396110 | *Myxas glutinosa* isolate IRLC |
| MH189886 | *Peregriana dolgini* voucher Mlym50 |
| KP242390 | *Radix labiate* isolate BZY7 |
| JN614409 | *Stagnicola palustris* isolate Lpal3 |
| HG932241 | *Lymnaea taurica* voucher SNSD Moll S2922 |
| MH189887 | *Lymnaea stagnalis* voucher Mlym51 |
| GU680908 | *Lymnaea stagnalis* voucher BIOUG<CAN>:09PROBE |
| MW221941 | *Lymnaea stagnalis* mitochondrion, |
| JN614411 | *Lymnaea stagnalis* isolate LstgG |
| NC_042905 | *Pseudosuccinea columella* isolate LS3 |
| JN051375 | *Lymnaea diaphana* haplotype L.dia |
| KT781292 | *Galba cubensis* isolate USA_NeB2_01 |
| AM494010 | *Lymnaea viatrix* ventricosa |
| AY227367 | *Galba bulimoides* |
| KT781297 | *Galba cubensis* isolate ARG_Canz_01 |
| AM494009 | *Bakerilymnaea cubensis* |
| FN182197 | *Lymnaea humilis* |
| JN614389 | *Galba cousin* isolate LCM2 |
| FN598161 | *Lymnaea cousini* haplotype L.cous.*cox*1-a |
| MN601407 | *Galba truncatula* voucher UGSB 23475 |
| KP242470 | *Galba truncatula* isolate DAS10 |
| JF461487 | *Galba truncatula* haplotype b |
| KP242688 | *Galba truncatula* isolate LYC15 |
| KM612176 | *Fossaria parva* voucher 08BBMOL-0093 |
| MN601409 | *Galba mweruensis* voucher UGSB 12151 |

**Supplementary table S2**. The updated nomenclature used in the phylogenetic tree of the current study and the previous species names corresponding to each of the GenBank Accession numbers

| Accession number | Species name | Updated species name |
| --- | --- | --- |
| HQ330989 | *Radix balthica* | *Ampullaceana balthica* |
| JN564796 | *Galba pervia* | *Orientogalba ollula* |
| KP098541 | *Radix balthica* | *Ampullaceana balthica* |
| NC 018536 | *Galba pervia* | *Orientogalba ollula* |
| AF485647 | *Ampullaceana balthica* | *Ampullaceana balthica* |
| AF485648 | *Radix luteola* | *Racesina luteola* |
| KY008521 | *Lymnaea diaphana* | *Pectinidens diaphanus* |
| JN872465 | *Galba viatrix* | *Galba viator* |
| HQ283243 | *Radix peregra* | *Peregriana peregra* |
| HQ283237 | *Lymnaea cousini* | *Galba cousini* |
| NC054237 | *Ampullaceana lagotis* | *Radix plicatula* |
| HQ283239 | *Lymnaea viatrix* | *Galba viator* |
| EU556225 | *Austropeplea tomentosa* | *Austropeplea brazieri* |
| EU556226 | *Austropeplea tomentosa* | *Austropeplea brazieri* |
| EU556227 | *Austropeplea tomentosa* | *Austropeplea brazieri* |
| EU556228 | *Austropeplea tomentosa* | *Austropeplea brazieri* |
| EU556229 | *Austropeplea tomentosa* | *Austropeplea brazieri* |
| EU556230 | *Austropeplea tomentosa* | *Austropeplea brazieri* |
| EU556232 | *Austropeplea tomentosa* | *Austropeplea brazieri* |
| EU556233 | *Austropeplea tomentosa* | *Austropeplea brazieri* |
| EU556240 | *Austropeplea tomentosa* | *Austropeplea subaquatilis* |
| EU556242 | *Austropeplea tomentosa* | *Austropeplea subaquatilis* |
| EU556243 | *Austropeplea tomentosa* | *Austropeplea huonensis* |
| EU556244 | *Austropeplea tomentosa* | *Austropeplea huonensis* |
| EU556245 | *Austropeplea tomentosa* | *Austropeplea huonensis* |
| EU556248 | *Austropeplea tomentosa* | *Austropeplea* cf. *brazieri* |
| EU556261 | *Austropeplea lessoni* | *Bullastra lessoni* |
| AY577463 | *Lymnaea glabra* | *Omphiscola glabra* |
| MG421709 | *Fossaria exigua* | *Galba exigua* |
| HQ330989 | *Radix balthica* | *Ampullaceana balthica* |
| KP242633 | *Radix balthica* | *Ampullaceana balthica* |
| EU818820 | *Radix relicta* | *Ampullaceana relicta* |
| NC018536 | *Galba pervia* | *Orientogalba ollula* |
| LC360950 | *Austropeplea ollula* | *Orientogalba ollula* |
| KP242390 | *Radix labiata* | *Peregriana labiata* |
| JN051375 | *Lymnaea diaphana* | *Pectinidens diaphanus* |
| AM494010 | *Lymnaea viatrix* | *Galba viator* |
| AM494009 | *Bakerilymnaea cubensis* | *Galba cubensis* |
| FN182197 | *Lymnaea humilis* | *Galba humilis* |
| FN598161 | *Lymnaea cousini* | *Galba cousini* |
| KM612176 | *Fossaria parva* | *Galba parva* |
